# A Letter from the Front

**Published:** 1918-12

**Authors:** 


### A Letter From the Front.

A thrilling account of Red Cross activities in France is told by Dr. Ethel Lyon Heard of Galveston, on duty as a Red Cross physician over there, in a letter to Mrs. George D. Morgan, superintendent of women's work and supplies of the local chapter of the Red Cross.

The letter follows:

Maternite Anglaise, Chalons-sur-Marne, June 11, 1918.—My Dear Mrs. Morgan: I was so glad to receive your welcome and interesting letter. News from Galveston and friends is always such a pleasure to me, and especially inside news from anything in which I continue to feel as great an interest as I do in the Red Cross there. I am proud of our chapter. I think it's a bully chapter and that the women of Galveston are perfectly splendid to work so faithfully and so sustainedly.

A great many interesting things have befallen since I wrote you last. In the first place, to go back a long time, when the big offensive began in the Somme, I was simply consumed with anxiety about Dr. Heard, whose battalion I knew was directly in the middle of the push. There were ten black days when I didn't have a line or a word from him, and when I finally got a field card laconically informing me that he was perfectly well, my spirits sprang to as great a height above the mean level as they had been depressed below it those nights when I listened to the guns and wondered where and how he was. From that time on I continued to get a line at intervals. When I think what I went through in two weeks, and that the women of France and England have been enduring that sort of thing and worse for four years, it is perfectly unbelievable.

I received a telegram from Dr. Heard saying for me to come to Rouen, where he was in the hospital. You can imagine I wasted no time in preparations. Fortunately, things were in such shape that I could safely leave for awhile, and by the kind offices of an angel of a French officer who occupies himself with the affairs of the Red Cross personnel in Chalons, I got my "*sauf conduit*" for Rouen in record time and walked into Dr. Heard's room at "No. 2" (British Red Cross Hospital) just twenty-four hours after I'd received his wire. He was already much better, having either been lightly gassed or else gotten a very bad bronchitis—it is hard to tell which.

VISITS CITY OF ROUEN.

I spent a very delightful twelve days in Rouen, in the course of which we saw many very beautiful and interesting things—the churches and monuments are not only beautiful in themselves, but full of historic interest. Especially the things connected with the trial and execution of Jeanne d' Arc were of great interest to me; the personality and life of "La Pucelle" seem especially appealing now, when the France she loved so much is again menaced and suffering. Dr. Heard remained in the hospital for about two weeks after I was obliged to leave him to return to my own job, and then went on duty again—at a base hospital this time—where he is a little less likely to come into disastrous countact with shells or bombs than when he was in the line. I don't suppose things have reached the point at home yet where one must produce a bread ticket before he can have any bread with a meal. As soon as I got to Rouen I went to the authorities with my certificate from the mayor of Chalons and got myself a bread ticket. Dr. Heard, being a patient in a hospital, was not entitled to a bread ticket, and consequently when we wanted to take a meal together at a restaurant or hotel he had to bring his ration of bread with him from the hospital! Quaint, isn't it?

When I returned to Chalons I found everything going on nicely, and soon after Miss Pye, the directrice of the place, went to England for her well-earned holiday. A few days after her departure the new offensive toward Paris began, and we found ourselves uncomfortably near the zone of operations. The cars were all busy all day helping to move the refugees, who of course fell back in a flood before the sudden advance of the enemy. Having word from the Red Cross commander of this zone that there was great need of help in the evacuation hospital at a neighboring town quite close to the area of battle, I took a nurse and my invaluable little Ford and went to see what we could do to help. On the roads one passed each moment convoys of refugees, all their possessions packed in a cart or on a hay wagon—goats, geese, chickens, sheep, cows and calves, loads of farm implements—as well as crowds of less fortunate ones on foot, carrying their few little clothes and bundles on their backs or in a wheelbarrow.. The relief workers of the Societe des Amis were already establishing aid posts at crossroads and in villages where there was most passing, giving hot soup and coffee and food to the refugees, and also helping with clothes when necessary and arranging means of transport to the nearest base for emigrant trains.

### Describes Evacuation Hospital.

Mlle. Merle, my splendid French nurse, and I went to the evacuation hospital—a large group of wooden barracks close to the railroad in an important town. This type of hospital is designed to receive wounded brought in from the first dressing stations at the front in ambulances to redress the wounds, operate if necessary, feed them and put them in trains for transportation as soon as possible to base hospitals. When we got there we found literally thousands of wounded. Stretchers stood on the ground wherever there was a piece of ground big enough to hold one. Wounded sat or lay on every available inch of space, and an unending stream of ambulances continued to flow in with wounded on the running boards, on the fenders —wherever they could stick on. We couldn't find anyone to ask what we should do, so we simply began and very soon orderlies or the girls from the cantine, who were washing faces and giving drinks and food, began to come and say, "There is a man badly off in D," or, "Could you come over to No. 12? There is a man there who hasn't been dressed for a week." We worked steadily all day, taking a few minutes off for some bread and a bite of chocolate. Toward the middle of the afternoon we discovered that nearly the whole staff of the place had had orders to leave and were gone and that there were no doctors there except one or two newly arrived from somewhere else who were utterly ignorant of where anything was or how to find their way about. By that time, having finished all the more urgent work we found in the various barracks we went to the central pavilion, where was the main dressing room and the room supposed to receive incoming cases; the latter, however, had long ago overflowed into all the space around the building, in spite of the fact that trains were being loaded as rapidly as they could be gotten. The whole thing was indescribably dreadful. Men in every imaginable state of injury, men having hemorrhages, men dying on every side; ambulances with four dead out of six they were carrying. The dressing room in a state of awful confusion—blood and bloody dressing and clothes that had been cut off and empty tetanus anti-toxin bottles and odds and ends all over everywhere.

### Treats Wounded Soldiers.

We worked side by side with the French doctor—medical officer—in that dressing room, giving anti-tetanic serum, dressing the cases that required it, trying to see that they had food and drink, or morphine or stimulants, as was necessary. I did dressings and put up wounds I never should have dreamed of touching without an operating room and an anesthetic and all sorts of aids of that sort, because they had to be done. To complicate matters, as soon as it got dark a violent air raid began, which made it necessary to put out the lights, and we continued our work by the light of a pocket electric lamp that Mlle. Merle had had the presence of mind to bring. Along about 2 a. m. the aerial bombardment was so severe that the ambulances stopped coming, since it was so dangerous to pass through the streets, and so by and by we found that all the cases there in rock from some bomb which struck nearby—and not caring a pin. We slept a few hours and went back to the hospital—to find again fresh floods of wounded, more and more work to do. We kept at it without interruption, having also a number of civilians who had been more or less severely injured by the air raid, until about 1 p. m. the word came that no more wounded were to be brought to this hospital and that the place was to be evacuated as soon as possible. We stayed until the last man was dressed—a poor fellow with a bullet through each upper arm, both broken, and the back of one hand shot away. I put up both his arms in splints, dressed his hand anew, and took off the tourinquet that was torturing him, gave him some milk and a hypo of morphine, and sent him on infinitely more comfortable than he had been in coming. One very comforting aspect of the whole affair is that nearly every case had been adequately handled at the first dressing station.

### Men Display Wonderful Patience.

And I will never, never, never forget the uncomplaining, stoical patience of those men—French or English; they did not utter one word of complaint; they were pathetically grateful for all one did for them; they died in silence, almost—those that did die—and they endured the unavoidable pain of redressing their injuries without a murmur. There were lots of French negro colonials among them, who were equally as admirable as the whites in that respect. I could have wept a river of tears over them if I had had time to weep. As it was, I shall never cease to be thankful that at that particular time of probably unparalleled emergency I could be there to do what little I could to help. It was a forty-eight hours I shall never forget. When all we could do was finished we got into Marie Anne (the invaluable Ford's name), and reporting at the American Red Cross office that the hospital was to be evacuated and that we would come back the next day to make sure we were not needed there or elsewhere, we flew back to Chalons, decidedly worried for fear, when so many bombs were dropping from the sky, some had fallen there. We found everything tranquil, however, to my relief.

The next day we went again; found on reporting to Red Cross headquarters that the hospital was already empty, as a perfectly equipped hospital train had come in and taken every blesse. That made me feel good. We had a rumor that at Sezanne, a town some forty kilometers to the south, there was also a bad congestion of wounded. So we went at once to Sezanne, only to find—O vagaries of military central offices and red tape!—that while at Sezanne there was a marked congestion of medical staffs, nurses and orderlies, at Sezanne there were no wounded coming in! At Montmirail, further on, we found many wounded, but plenty of people to care for them, so we reported back to Red Cross headquarters and came home, satisfied the situation which had existed because of numbers of wounded and some confusion of orders no longer existed—thank heaven! The advance was stopped by then and the number of casualties greatly reduced.

### Refugees Come From Chalons.

We soon found enough to keep us thoroughly occupied at home, for the refugees began to come by Chalons—caisson loads of women and children brought to this place to stay a day or so until a trainload was ready—cases brought in for our maternity almost invariably accompanied by a brood of small children, which must be fed, and sheltered and often clothed, and all too often cleaned—sick children picked up in the villages.

We are busy all day every day, trying to supply the actual needs of the emigrants in the shape of baths, clothing, food for the tiny children, as well as our regular work, and since we are full to our utmost capacity of ninety-six beds, time does not hang idle on our hands. Furthermore, we are in the act of scouring the Aube for a suitable building to which to evacuate in case a push directly in our direction renders Chalons uninhabitable, which may happen any time, or may not happen at all. I certainly devoutely pray it won't happen, for anything more awful to contemplate than transferring the patients, babies, children, beds, chairs, tables, staff, bed linen, food, help, drugs, etc., of this place to some other part of France I can not imagine, especially as the people are in the beds at the present moment. However, if it's a choice between that and shells, followed by Germans, I shall probably be quite willing to go.

I hope the censor decides to let this go through as it is. I don't often find time to write a long letter like this and I should hate to have it chopped up. I sort of depend on you to give my news and greeting to the innumerable people to whom I ought to write, and haven't, and tell them it's simply because I haven't time. I haven't even sent a report to Paris for weeks. I must say a word about how good people at home have been about sending me baby clothes and children's clothes. And they have all been so useful! Do you know Mrs. Tonella? She has sent fifteen packages of clothes—the old dear. I am sorry your bag didn't turn up. I will try again. I only wish we could send large amounts of the broiderie to America; it is made by refugee women as one of the branches of the relief work of the Societe des Amis, but the duty is 60 per cent, so I am afraid it wouldn't pay.

Please give my best regards to all my friends, and with very best wishes for yourself and congratulations on your good work there, I am yours faithfully.—Galveston News.

---

The *Medical Review of Reviews* is to absorb the Buffalo Medical Journal, beginning with its January, 1919, issue. This is the third publication which the *Review* has purchased during the past few years.

---

The *Medical Review of Reviews* further announces that it will be greatly increased in size beginning with the January, 1919, issue but that the subscription price is not to be increased.

---

Brown would never trust his grandfather's clock to the movers, but always carried it himself, until one day a very puzzled passerby stopped him and said, "Say, mister, I just wanta ask you a question. Why don't you get a watch?"

